# Supplementary material for: Protocol for the Swiss COhort of Healthcare Professionals and Informal CAregivers (SCOHPICA): Professional trajectories, intention to stay in or leave the job and well-being of healthcare professionals
Source: PLoS One. 2024 Aug 29;19(8):e0309665. doi: 10.1371/journal.pone.0309665 (PMC11361676; doi:10.1371/journal.pone.0309665)
Supplement: Attachment — Submitted filename: Answers to Reviewers_2024_04_26.pdf [file pone.0309665.s002.pdf]

Response to the Reviewer #2 comments

Introduction

- It might be interesting to incorporate briefly into the introduction some of the European Union's efforts to address job retention in healthcare, and to situate your research in this framework. For instance, the European Union has actively engaged in mitigating this matter by implementing multiple initiatives since 2012, such as the Joint Action Plan (JAP) for the EU Health Workforce (2013–2016) or establishment of the Support for the Health Workforce Planning and Forecasting Expert Network (SEPEN):
  - Kroezen, M., Van Hoegaerden, M., & Batenburg, R. (2018). The Joint Action on Health Workforce Planning and Forecasting: Results of a European programme to improve health workforce policies. Health Policy, 122(2), 87–93. https://doi.org/10.1016/j.healthpol.2017.12.002
  - Directorate-General for Health and Food Safety. (2023). Public Health Overview. https://health.ec.europa.eu/health-workforce/overview_en#sepen---support-for-the-health-workforce-planning-and-forecasting-expert-network-2017--2018

  Our response: We thank the reviewer for this suggestion. We have now added these elements in the introduction; changes read as follows in the manuscript (line 72 to 81):

  "The issue of HCPs' retention has been raised as a concern for over a decade (4). Since 2012, the European Union has launched several programs, such as the Join Action Plan (5) and the Support for the Health Workforce Planning and Forecasting Expert Network, (6) aimed at improving the retention of HCPs and addressing their shortage. In Switzerland, efforts have been made by health departments of the universities of applied sciences, which collaborated to create the Competence Network Health Workforce. This network aims to define a national strategy to tackle shortages of HCPs. At the political level, the Swiss population approved a constitutional law in 2021, compelling Swiss cantons and the federal government to ensure the sufficient availability of qualified nurses, and therefore to collect data to monitor the implementation of this new law (8). "

- You write 'Life history calendar (LHC), a tool designed to collect retrospective data from participants by maximizing their possibilities of recalling past events completely and accurately (18, 19), has not been used in health workforce research yet, despite having been successfully used in different contexts.' I am not familiar with this tool, but I am very interested. It might benefit the reader to briefly mention the contexts in which it has been successfully used.

  Our response: We agree with the reviewer that it is indeed appropriate to mention some contexts in which LHCs have been used. We have added sentences to specify contexts in which LHCs have been applied successfully, changes appear in the text as follows (line 101 to 106):

  "LHCs have proven successful across various contexts, including studies on the trajectories of unemployed and vulnerable individuals, the sexual life of young people (25, 26), and in general population surveys (27). These studies have shown that 1) LHCs are more efficient than traditional sociodemographic questions for collecting retrospective data; 2) the data collected is reliable (28); and 3) online versions of LHCs can be used to reach large samples of participants (29, 30)."

- Consider adding a fourth area for further research, focusing on the inclusion of various healthcare settings (beyond hospitals or general practices), as this will clearly be a benefit from your study.

Our response: The suggested area has been added in the manuscript, and changes appear in lines 118, 119, as follows: *"Finally, it is key to investigate settings beyond the two most frequently studied, namely hospitals and general practices. "*

- Regarding the media's coverage of deteriorating working conditions and staff shortages among healthcare workers, it may be advisable to delete this information, as media reports may not always be reliable sources for such data.

Our response: We have removed the mention of media, as suggested.

- In the section about Switzerland, consider shortening the content and including more international studies on the topics to better address the article's international audience.

Our response: We have modified the text as proposed. Information about the Swiss context has been shortened and we have added information on the international context with the example of several countries. The whole section reads now as follows (line 121 to 144):

"*In Switzerland, the deteriorating working conditions of HCPs and staff shortages have been stressed by scientific studies and reports for several years, and this situation has worsened since the COVID-19 pandemic. Reports from the Swiss Health Observatory have predicted that a large number of HCPs would need to be hired to meet population needs, and that physicians' supply in the ambulatory sector would not be sufficient by 2030 (42- 45). Additionally, a recent report has highlighted that 70,000 nursing staff will be needed by 2029, which encompasses both workforce replacements needs and the increased demand for additional staff stemming from population healthcare needs (46). In fact, the coverage rate is predicted to be lower than 80% with a clear deficit between workforce supply and projected needs (46, 47). This situation mirrors an international trend. Indeed, the WHO has projected a shortage of 15 million HCPs by 2030. In Germany, for example, estimates for the required number of HCPs in 2030 ranged from approximately 263,000 to nearly 500,000 full-time equivalents. (48) Similarly, as of September 2023, the UK's National Health Service (NHS) reported 121,000 full-time equivalent vacant positions. (49) Finally, several reports have indicated that the United States will face a shortage of up to 124,000 physicians by 2033 and will require 200,000 nurses annually to meet the increasing care demand. (50) As in other countries, Swiss healthcare stakeholders have stressed the paucity of data, hindering effective monitoring, planning, and managing of the health workforce. Research projects aimed at both collecting data and leveraging HCPs retention have also been conducted in the Swiss context. These have investigated job stress, job satisfaction, burnout, and intention to leave the job/profession (51-65). However, like studies conducted in other countries, these publications mostly concentrated on nurses and physicians (mainly in hospital setting), and both nationwide and longitudinal data across multiple healthcare sectors are lacking to understand HCPs' professional trajectories, well-being, and intention to stay in/leave their position/profession/health sector.*"

- The part concerning informal caregivers might be better situated within the study design rather than the introduction.

Our response: We agree with the reviewer; the section has been moved to the methods section (Study design sub-section, current lines 170 to 173).

Methods and materials

- Sample size:

- Quantitative: ‘For the first baseline survey (fall 2022), 1500 HCPs were targeted.’ -> This was in fall 2022, this means you reached that target? And do you mean 5 to 10.000 unique participants?

  Our response: In the cohort, we target to follow at least 5000 to 10000 unique HCPs, in the medium term. As the cohort is an open cohort, which means that recruitment will be conducted every year, we expect this number to be achieved progressively. The target for the first recruitment was 1500. Upon reviewing the sentence you referenced, we acknowledge that mentioning the 1500 first participants may be unclear. Hence, the sentence has been clarified; it now reads as follows (line 189 to 190):

  “*In the quantitative phase, SCOHPICA aims to collect data from 5,000 to 10,000 unique baseline HCPs, whom we will follow over the years.”*

- Qualitative :
  - ‘Participants who agreed to be contacted in the baseline survey will be invited for individual or group sessions.’ In the event of a low response rate, will you continue open recruitment or adhere to those that agreed to be contacted in the baseline survey?

    Our response: Thank you for this remark. Regardless of the response rate, participants included in the qualitative phase will be selected from the different recruitment conducted from 2022. Even a low response rate for the qualitative phase (for example 10%) would lead to include 500 HCPs in a qualitative study, considering the anticipated 5000 participants in SCOHPICA. This would be sufficient to identify participants for the qualitative phase of the study.

  - What is your rationale for eight HCPs? Any reference or guidelines you are referring to?

    Our response: There are no clear guidelines concerning the number of participants to include in concept mapping sessions and reviews are quite heterogeneous regarding this issue. Usually, concept mapping involves between 20 to 30 participants. However, some study with specific objectives have reported including fewer than 10 participants. It is well established that the number of participants is mainly linked to the characteristics of participants and the topic of the study, with careful consideration of the interaction between the two factors. Challenges in participant recruitment, as well as the expertise of participants regarding the topic of the study may justify smaller participant numbers in concept mapping sessions. In our case, concept mapping will be conducted with HCPs from different professions with varying work schedules, across different care contexts and cities throughout Switzerland. This makes it difficult to gather large numbers of HCPs for a single session. Therefore, we have pragmatically set the number of participants per session at eight. Although this number may be considered low compared to other studies using concept mapping, it will be compensated by conducing multiple concept mapping sessions, resulting in the inclusion of 120 participants in this qualitative phase. However, since not every aspect of the qualitative phase can be predetermined at this stage, we reserve the flexibility to adjust the number of participants as needed. This has been added in the article, which now reads as follows (lines 205-206): “*according to the*

characteristics of participants, the chosen topic, the specific method and data saturation assessment".

We also added a reference (line 206).

- Recruitment:
  - Will recruitment efforts be continuous or periodic? When precisely will the survey be open each year: periodically or throughout the year?

    Our response: The recruitment will be periodic and last four months every year. This has now been specified in the manuscript (lines 197 to 198):

    "*Since SCOHPICA is an open cohort, new participants will be recruited every year between October 1st and January 31 of the following year*."

  - Why limit the project presentation to Switzerland? Why not present it at international conferences? Many international conferences could offer a valuable platform for your study.

    Our response: The presentations you mention are those planned for the recruitment of HCPs (section 2.2.2. recruitment of healthcare professionals). These are part of the communication strategy for the recruitment of participants, which is only conducted in Switzerland (i.e., inclusion criteria: working in Switzerland).

- Data collection:
  - Quantitative:
    - 30 minutes is a long time, have you considered omitting some questions to avoid high dropout rates? Will there be any follow-up or reminders for people who do not complete the survey until the end? Additionally, if I understand correctly, participants do not have to register beforehand?

      Our response: Actually, we have already reduced the initial length of the questionnaire to focus only on the determinants of the intent to stay most frequently reported in the literature. Sociodemographic and socio-professional questions comprise a large part of the baseline questionnaire, yet some of these will not be included in the follow-up questionnaire, resulting in shorter follow-up questionnaires.

      For the baseline questionnaire, we are not able to send reminders as we do not have HCPs' email addresses. However, during the follow-up surveys, we will contact baseline participants who provided their contact details (i.e. email address), and send up to three reminders to those who did not respond to the questionnaire.

      Following data collection each year, we will assess missing data and consider shortening the questionnaire if we observe that some questions are systematically skipped, for example.

    - I would also suggest to briefly mention which validated measurement scales will be used in the text (e.g. copsoq, etc), instead of only listening them in a table. The table can be used for detailed explanations and a comprehensive overview.

Our response: As suggested, we now mention the name of the scales in the manuscript and not only in Table 1.

Changes appear as follows (lines 276 to 297):

"We chose the following validated scales to measure these determinants (see details in Table 1):
- *Quantitative workload inventory (69): workload.*
- *Copenhagen Psychosocial Questionnaire (COPSOQ; 70): control over working time; possibilities for development; work-life conflict; influence at work; sense of community at work; meaning of work; job satisfaction.*
- *Practice Environment Scale of the Nursing Work Index (PES-NWI; 71): staffing and resources.*
- *Global transformational leadership scale (72): transformational leadership.*
- *Intensity of interprofessional collaboration (73): interprofessional practice.*
- *Recognition at work scale (74) : recognition from managers, colleagues and patients.*
- *Rushton moral resilience scale (75).*
- *Intolerance of uncertainty scale (76).*
- *The one-item MBI:EE (77): non-proprietary single-item of burnout.*
- *Self-rated health single item (78).*
- *Organizational Support Subscale of the Nursing Work Index (79): perceived quality of care.*

Four not-validated items were drawn from a study of Shanafelt and colleagues (80) to complement the assessment of perceived quality of care. Moreover, two questions were developed by the research team to assess "Preparedness to work reality" (as no validated scale is currently available for this construct). We conducted a qualitative pre-test of the questionnaire with 20 participants from diverse backgrounds."

- Furthermore, I recommend clustering all the occupation-related aspects in the text to ensure a clear overview, while elaborating on them (i.e., all categories) in a table format. For example, mentioning all the different income categories in the manuscript is unnecessary; it would be more suitable to include them in a table or supplement.

Our response: The text has been shortened and details appear now in a new table (Table 2). Text now appears as follows (lines 298 to 310):

"Additionally, the baseline questionnaire collects information on respondents' work and occupation-related aspects (Table 2).

Socio-professional information included: employment status, professional status, employment rate, occupational context, current profession, training and specialization, additional training, years of professional experience, main occupational domain, hours worked per day, days worked per week, type of work shift, type of work schedule, managerial responsibilities, country of education, other past profession(s), modification of employment rate in past 12 months, number of employers/occupational context/job location changes, since starting to work in healthcare, career interruption(s) of one year or longer, unemployment period(s), work-related accident(s)/sick leave(s), healthcare sector throughout career, healthcare sector of first employment, work location, and commuting time to work.

Sociodemographic information included: gender, year of birth, nationality, marital/partnership status, children, informal caregiving status, monthly individual incomes, monthly household incomes, and residency location."

- Qualitative:
  - Will interviews and focus groups be conducted exclusively face-to-face, or will there be an option for online participation? Please discuss the advantages and disadvantages of both approaches and provide insight into the rationale behind your decisions.
    - E.g. Boone, A., Braeckman, L., Michels, N., Kindermans, H., Van Hoof, E., Van den Broeck, K., & Godderis, L. (2023). Accessibility, Retention and Interactivity of Online Co-Creation Workshops: A Qualitative Post-Hoc Analysis. International Journal of Qualitative Methods, 22. https://doi.org/10.1177/16094069231180169

    Our response: We thank the reviewer for this suggestion and the reference. Focus groups and interviews will probably take place face-to-face and online. The choice will be made according to the context and other factors such as availability of participants, possibility to remunerate participants and/or travels. We cannot specify, currently, which strategy will be applied. We will be able to discuss this in forthcoming articles focusing on qualitative results. We thank the reviewer again for the reference which we noted and will refer to in upcoming articles.

  - Who will be responsible for moderating the sessions? If multiple moderators are involved, how will you ensure consistency throughout the sessions?

  Our response: The same two moderators will conduct all the sessions within each linguistic region. To ensure consistency between linguistic regions, regular meetings will be organized between the teams. We will adhere to qualitative method standards for conducting interviews and focus groups. We thank the reviewer for bringing up this point, which we will mention in our future papers on qualitative analyses and results.

  - Could you elaborate on how data saturation will be assessed in the qualitative part?

    Our response: We have added a reference to data saturation into the text (line 206). As we will conduct concept mapping, saturation will be easily observed through the different maps generated. Saturation will be considered achieved when no new elements emerge in the map. To assess saturation, we will use the procedure proposed by Guest, Namey and Chen (2020), which is now referenced in the text (line 206). Since the manuscript presents the protocol and we have not yet performed this assessment, we will not describe it into further details in this article. The procedure for assessing data saturation will be outlined in future articles presenting qualitative analyses and results.

- Ethics:
  - Will participants need to provide separate informed consent for each instance of participation? Are there distinct forms for the qualitative and quantitative components of the study?

Our response: At baseline, participants provide consent for the entire project and future use of data (these two independent consents were required by the ethics committee). A new ethics approval will be necessary for the qualitative part, as well as a specific informed consent. This is now specified in the manuscript as follows:

"*At baseline, before accessing the electronic questionnaire..." (430 - 431)*

"This double consent is valid for the quantitative part of SCOHPICA; for the qualitative part, a specific informed consent will be required." (437 – 438)

"A separate ethics committee approval will be necessary for the qualitative phases of SCOHPICA." (448 – 449)
