# Supplementary material for: Protocol for the Swiss COhort of Healthcare Professionals and Informal CAregivers (SCOHPICA): Professional trajectories, intention to stay in or leave the job and well-being of healthcare professionals
Source: PLoS One. 2024 Aug 29;19(8):e0309665. doi: 10.1371/journal.pone.0309665 (PMC11361676; doi:10.1371/journal.pone.0309665)
Supplement: Attachment — Submitted filename: Peer Review Report.docx [file pone.0309665.s001.docx]

Dear Author(s),

Thanks a lot for your relevant and interesting protocol. The topic is highly important and we are happy to see that this study will happen in the near future. Below you can find a few comments to give more strength to your protocol.

Introduction

- It might be interesting to incorporate briefly into the introduction some of the European Union’s efforts to address job retention in healthcare, and to situate your research in this framework. For instance, the European Union has actively engaged in mitigating this matter by implementing multiple initiatives since 2012, such as the Joint Action Plan (JAP) for the EU Health Workforce (2013–2016) or establishment of the Support for the Health Workforce Planning and Forecasting Expert Network (SEPEN):
  - Kroezen, M., Van Hoegaerden, M., & Batenburg, R. (2018). The Joint Action on Health Workforce Planning and Forecasting: Results of a European programme to improve health workforce policies. Health Policy, 122(2), 87–93. <https://doi.org/10.1016/j.healthpol.2017.12.002>
  - Directorate-General for Health and Food Safety. (2023). Public Health Overview. <https://health.ec.europa.eu/health-workforce/overview_en#sepen---support-for-the-health-workforce-planning-and-forecasting-expert-network-2017--2018>
- You write ‘Life history calendar (LHC), a tool designed to collect retrospective data from participants by maximizing their possibilities of recalling past events completely and accurately (18, 19), has not been used in health workforce research yet, despite having been successfully used in different contexts.’ I am not familiar with this tool, but I am very interested. It might benefit the reader to briefly mention the contexts in which it has been successfully used.
- Consider adding a fourth area for further research, focusing on the inclusion of various healthcare settings (beyond hospitals or general practices), as this will clearly be a benefit from your study.
- Regarding the media's coverage of deteriorating working conditions and staff shortages among healthcare workers, it may be advisable to delete this information, as media reports may not always be reliable sources for such data.
- In the section about Switzerland, consider shortening the content and including more international studies on the topics to better address the article's international audience.
- The part concerning informal caregivers might be better situated within the study design rather than the introduction.

Methods and materials

- Sample size:
  - Quantitative: ‘For the first baseline survey (fall 2022), 1500 HCPs were targeted.’ -> This was in fall 2022, this means you reached that target? And do you mean 5 to 10.000 unique participants?
  - Qualitative :
    - ‘Participants who agreed to be contacted in the baseline survey will be invited for individual or group sessions.’ In the event of a low response rate, will you continue open recruitment or adhere to those that agreed to be contacted in the baseline survey?
    - What is your rationale for eight HCPs? Any reference or guidelines you are referring to?
- Recruitment:
  - Will recruitment efforts be continuous or periodic? When precisely will the survey be open each year: periodically or throughout the year?
  - Why limit the project presentation to Switzerland? Why not present it at international conferences? Many international conferences could offer a valuable platform for your study.
- Data collection:
  - Quantitative:
    - 30 minutes is a long time, have you considered omitting some questions to avoid high dropout rates? Will there be any follow-up or reminders for people who do not complete the survey until the end? Additionally, if I understand correctly, participants do not have to register beforehand?
    - I would also suggest to briefly mention which validated measurement scales will be used in the text (e.g. copsoq, etc), instead of only listening them in a table. The table can be used for detailed explanations and a comprehensive overview.
    - Furthermore, I recommend clustering all the occupation-related aspects in the text to ensure a clear overview, while elaborating on them (i.e., all categories) in a table format. For example, mentioning all the different income categories in the manuscript is unnecessary; it would be more suitable to include them in a table or supplement.
- Qualitative:
- Will interviews and focus groups be conducted exclusively face-to-face, or will there be an option for online participation? Please discuss the advantages and disadvantages of both approaches and provide insight into the rationale behind your decisions.
  - E.g. Boone, A., Braeckman, L., Michels, N., Kindermans, H., Van Hoof, E., Van den Broeck, K., & Godderis, L. (2023). Accessibility, Retention and Interactivity of Online Co-Creation Workshops: A Qualitative Post-Hoc Analysis. International Journal of Qualitative Methods, 22. <https://doi.org/10.1177/16094069231180169>
- •Who will be responsible for moderating the sessions? If multiple moderators are involved, how will you ensure consistency throughout the sessions?
- Could you elaborate on how data saturation will be assessed in the qualitative part?
- Ethics:
  - Will participants need to provide separate informed consent for each instance of participation? Are there distinct forms for the qualitative and quantitative components of the study?

Despite these comments, many thanks for your hard work and good luck with the research!

Regards
