# Supplementary material for: Protocol for the Swiss COhort of Healthcare Professionals and Informal CAregivers (SCOHPICA): Professional trajectories, intention to stay in or leave the job and well-being of healthcare professionals
Source: PLoS One. 2024 Aug 29;19(8):e0309665. doi: 10.1371/journal.pone.0309665 (PMC11361676; doi:10.1371/journal.pone.0309665)
Supplement: Attachment — Submitted filename: 20240724_Peytremann-Bridevaux_Response to reviewers_R2.docx [file pone.0309665.s003.docx]

Response to Reviewer #3:

We would like to thank the Reviewer for taking the time to review our protocol. Please find our responses to the comments below here.

*Reviewer #3: The manuscript could be accepted after addressing minor comments. As of now, it looks good and authors sufficiently address comments of other reviewers in first stage of revision. An abstract should contain a potential implications.*

We agree that is it important to underscore the potential implications in the abstract. Therefore, we have made the following additions in bold. The modified Discussion section of the abstract now reads as follows:

Discussion: Using innovative methodologies, SCOHPICA will gather nationwide and longitudinal data on HCPs practicing in Switzerland. **These data could have numerous implications: promoting the development of research related to HCPs’ well-being and retention intentions; supporting the development of policies to improve working conditions and career prospects; contributing to the evolution of training curricula for future or current healthcare professionals; aiding in the development of health systems capable of delivering quality care; and finally, providing the general public and stakeholders with free and open access to the study results through an online dashboard.**

*The keywords chosen are not particularly appropriate. It is recommended to rewrite the keywords based on the content of this article.*

Many thanks for your feedback, we have amended the keywords as follows:

Healthcare professionals, career trajectories, well-being**, retention intentions, staff turnover,** cohort **study**, mixed methods

*The methods section should include the design and setting of the study and a clear description of all processes, interventions and comparisons*

We believe that the design and setting of the study, along with descriptions of all processes, measurements and analyzes, are thoroughly detailed in the Methods section of the manuscript. Please note that there are neither interventions nor specific comparisons in our study.

Response to Reviewer #4:

*Reviewer #4: Peytremann-Bridevaux et al. presented the manuscript study protocol Protocol for the Swiss COhort of Healthcare Professionals and Informal CAregivers (SCOHPICA): professional trajectories, intention to stay in or leave the job, and wellbeing of healthcare professionals. I’ve read the manuscript with great interest, the main messages are clear and could be interesting for the journal and a broader audience. The protocol fully describes all study procedures. It is a well-written manuscript and a well-prepared investigation.*

We thank the Reviewer for taking the time to review our manuscript and for the positive feedback, which is very encouraging. We are pleased to hear that that the Reviewer found the study protocol well-described and well-prepared.
